# Chitosan coating silver nanoparticles as a promising feed additive in broilers chicken

**DOI:** 10.1186/s12917-023-03826-7

**Published:** 2023-12-09

**Authors:** Eman I. Hassanen, Ahmed M. Hussien, Sally Mehanna, Eman A. Morsy

**Affiliations:** 1 Pathology Department, Faculty of Veterinary Medicine, Cairo University, P.O.Box 12211, Giza, Egypt; 2 Toxicology and Forensic Medicine Department, Faculty of Veterinary Medicine, Cairo University, Giza, Egypt; 3 Department of Veterinary Hygiene and Management, Faculty of Veterinary Medicine, Cairo University, Giza, Egypt; 4 Poultry Disease Department, Faculty of Veterinary Medicine, Cairo University, Giza, Egypt

**Keywords:** Broilers, Nanoparticles, Performance, Histopathology, Immunology

## Abstract

The present study aimed to evaluate the potential of chitosan coating silver nanoparticles to enhance the growth performance and immune status of broilers without inducing oxidative stress-related pathological lesions in any organs or leaving residues of silver in the edible parts. Five clusters of Cobb one-day-old chicks (n = 10/group in each replication) were given oral therapy, once a week for 36 days as follows: (1) distilled water, (2, 3) 0.5- and 5 ppm silver nanoparticles (AgNPs), respectively, (4, 5) 0.5- and 5 ppm chitosan/silver nanoconjugates (CS/Ag-NCs), respectively. The results demonstrated a marked elevation in the body weight gain with a decline in the food conversion ratio and marked improvement in feeding and drinking behavior of all nanoparticles treated groups, but higher in CS/Ag-NCs groups than AgNPs groups and control group. In contrast to the 0.5 ppm AgNPs receiving group, the group receiving 5 ppm AgNPs noticed remarkable histological changes in some organs, including the liver, kidneys, spleen, and heart. Moreover, the administration of CS/Ag-NCs at two dosage levels didn’t influence any histological changes. The AgNPs groups’ antibody titers against the ND and AI viruses were almost identical to those of the control group. Otherwise, CS/Ag-NCs groups recorded the highest antibody titers. Additionally, there was a significant increase in silver content in most edible organs of AgNPs groups at a dosage level of 5 ppm. Otherwise, the coating of AgNPs by CSNPs could decrease the aggregation of silver in the biological organs. Thus, we recommend utilizing 0.5 ppm CS/Ag-NCs in broiler farms to promote their growth performance and strengthen their immune defense.

**Supplementary Information:**

The online version contains supplementary material available at 10.1186/s12917-023-03826-7.

## Introduction

The intensive use of sub-therapeutic antibiotic dosage as a feed additive has been shown to improve feed efficiency and growth performance in poultry. However, this practice contributes to the development of antibiotic-resistant bacteria [1]. As a result, there is an increasing interest in the animal feed sector in looking at alternatives to antibiotic feed additives. Among the antibiotic alternatives that could be used are essential oils, probiotics, and prebiotics [2–4]. Additionally, metal nanoparticles such as zinc [5], copper [6], gold [7, 8], and silver [9, 10] are examples of potential antibiotic substitutes. Studies have suggested that feeding of mineral nanoparticles improved digestive efficiency, immunity, and performance in livestock and poultry [11].

Silver nanoparticles (Ag-NP) have a lot of interest because of their broad-spectrum antimicrobial capabilities [12], so they are used in a lot of biological and medical disciplines [13, 14]. They have higher antibacterial activity than other metals, such as mercury, copper, lead, and chromium [15]. Nevertheless, silver ion (Ag+) is cytotoxic [16], its use as a feed additive necessitates chelation, which involves converting the positive charge (Ag+) to a non-charge (Ag^0^) molecule. Several Ag-NPs-based composites have shown improved performance due to nanoparticle stabilization and support [17].

Chitosan-silver nanoparticles (CS/Ag-NPs) as silver-based nanocomposites represent an emerging group of bio-nanostructured hybrid materials owing to their biocompatibility and biodegradability [18]. Chitosan is a biopolymer made from the chitin found in shellfish exoskeletons [19]. It exhibits antibacterial and antifungal properties [20, 21], as well as excellent metal chelator potential [22]. Chitosan is a non-toxic material that is safe for humans, animals, and the environment. In the poultry field, chitosan can be used as a feed supplement [23]. Coating AgNPs with polymers as CSNPs significantly increases their antimicrobial efficacy and makes them used in food packaging, as it increases the shelf life of food products [24]. For the previous reasons, this study was conducted to figure out the role of Chitosan-silver nanoparticles as a feed additive in broiler chickens by measuring its impact on broiler performance, immune status, microscopic picture of muscles, and different organs. Additionally, the safety assessments of these nanoparticles were evaluated by measuring the content of silver in different musculature and edible parts.

## Materials and methods

### Preparation of silver nanoparticles

According to the procedure outlined by Moghaddam et al. [25], the colloidal solution of AgNPs at a concentration of 500 ppm was created by the co-precipitation method by the reduction of silver nitrate (AgNO3) (99.99%, Aldrich, US) with Sodium borohydride (99%, Aldrich, US).

### Preparation of Chitosan-silver nanocomposites

Chitosan-silver nanocomposites (CS/Ag NCs) were synthesized through chitosan reduction of silver nitrate in accordance with Babu et al. [26].

### Characterization of the prepared nanoparticles

A double-beam UV-Vis-NIR spectrophotometer (Cary 5000, Agilent, UK) operating in the scanning range of 300–800 nm was used to record the characteristic SPR of AgNPs using absorption spectroscopy. To evaluate the morphology of the produced NPs, a transmission electron microscope (TEM) equipped with a Tecnai G2 200 kV accelerating voltage (FEI, Netherlands) was employed. Using a Malvern, ZS Nano, U.K. zeta sizer, the average particle size distribution curve and zeta potential of both NPs were ascertained using the dynamic light scattering (DLS) approach. An X-ray diffractometer (XRD, X’Pert Pro, Analytical, Netherlands) approach was used to analyze the crystalline and phase structure of the produced nanoparticles.

### Animals and experimental study

All sections in the current study adhered to the ARRIVE guidelines for the in vivo animal experiments (PLoS Bio 8(6), e1000412,2010​) and reported in the ARRIVE checklist. The Institutional Animal Care and Use Committee at Cairo University (IACUC, approval number: Vet CU 03162023669), Cairo, Egypt, approved all procedures, which were conducted in accordance with the laboratory animal care and use guidelines (8th edition, 2011).

A local hatchery (El-Hwamdya, Giza, Egypt) provided a total of one hundred one-day-old Cobb broilers chicks of both sexes. The chicks were weighed and randomly divided into five groups, each of which contained two replicates of ten birds in each (n = 10) based on the data obtained from the sample size calculation formula, whereas the single bird is considered as the experimental unit. They were raised in a deep-litter system with straw bedding, and the house was kept at standard humidity, ventilation, and temperature levels throughout the experiment. It also had a 24-hour constant-light schedule. The birds were provided unrestricted access to water and a balanced meal (including starting and growth diets) that was suitable for their maturation stage. Additionally, the birds received vaccinations against ND (live Newcastle) and IBV (Hitchner IB) through the ocular route on the seventh day of age, SC administered the H5N1 (avian influenza reassorting inactivated vaccine) on the tenth day of age, and IBD (virus strain winter field) through the ocular route on the twelfth day of age.

The birds in each replication were randomly separated into five groups (n = 10/group) using a block randomization strategy. Group 1 served as the control group, receiving sterile normal saline orally once a week for 36 days. Groups (2) and (3) received 0.5 and 5 ppm of silver nanoparticles (AgNPs), respectively. Chitosan/silver nanoconjugates (CS/Ag NCs) were administered in concentrations of 0.5 and 5 ppm to Groups 4 and 5, respectively. Starting from 7 days, all chicks were orally administered the nanoparticles using a stomach tube, once a week for 36 days. AgNPs dosages were chosen in accordance with the results of the prior study [11], and the same doses were used for CS/Ag NCs since broiler chickens lack a reference dose for CS/Ag NCs. The experimenters were blinded to the therapy while processing data and making exclusion decisions. Throughout the experimental period, no adverse events were reported and therefore, we didn’t exclude any birds.

### Behavioral parameters

Following the acclimation period (7 days), each bird was observed for a total of 15 min by instantaneous scan sampling three days per week, twice per day, and the behaviors were measured as a percentage of the entire number of birds seen throughout an instantaneous scan sample [27, 28]. Feeding, drinking, leg, and wing stretching, and preening behaviors were all recorded.

### Growth performance assessments

The body weight and feed intake of 10 birds (5 from each replication) were measured weekly in each group in a blinded manner. Furthermore, the feed conversion ratio (FCR), was calculated according to Timmerman et al., as the ratio of feed intake to weight gain for each bird/week [29].

### Sampling

Weekly blood samples were taken from the wing vein of ten chicks (five chicks per replication) that were selected by an investigator who was unaware of the treatment groups. The collected blood samples were centrifuged for five minutes at 4500 rpm to obtain clear serum samples and then stored at -20 °C until required for biochemical measurements (hemagglutination inhibition test and oxidative stress evaluation). To get samples from the skeletal muscles and other organs, including the liver, kidneys, heart, spleen, thymus, and bursa of Fabricius, all birds were euthanized by the exsanguination at the end of the experiment by cutting their throat and allowing them to bleed out. For histopathological analysis, 10% neutral buffered formalin was used to fix the collected tissue samples.

### Hemagglutination inhibition test

The hemagglutination inhibition (HI) test was used to measure the anti-ND and anti-AI vaccination antibody titers in serum samples at 8, 15, and 22 days old to assess the impact of both NPs at two dosage levels on the humeral immunity. It was done on 10 birds per group (5 birds from each replication). Erythrocytes are added after the virus is treated with diluted serum. The maximum dilution of serum that suppresses hemagglutination is determined by the HI titer following incubation [30].

### Oxidative stress evaluation

At the end of the experiment, lipid peroxidation was evaluated by measuring the serum levels of malondialdehyde (MDA) following the method described by Ohkawa et al., [31]. Additionally, catalase (CAT) activity and total antioxidant capacity (TAC) were measured following the method reported by Aebi, [32], using commercial kits (Biodiagnostic Co., Cairo, Egypt).

### Histopathological examination

The formalin-fixed tissue samples were extracted using standard procedures, including an ascending alcohol grade for dehydration and xylene for clearing. Then, the formalin-fixed tissue samples were cut into 4-micron slices using an ordinary microtome and placed on slides for histopathological analyses using the H&E stain [33].

Following the procedures outlined by Hassanen et al., [9], the severity of the evident microscopic lesions was evaluated using the traditional semiquantitative multiparametric grading methodology. Seven microscopic fields per slide in a total of 10 slides per group were graded by a pathologist who was unaware of the treatment groups to avoid bias. The diffuse lesion was blindly evaluated and graded on a 5-point scale as mild, moderate, severe, and extensive severe changes as follows: (1) Mild 25%, (2) Moderate 25–50%, (3) Severe 50–75%, and (4) Extensive Severe > 75% tissue damage are all possible outcomes. Meanwhile, the focal lesions were graded as follows: according to the approach of Hassanen et al., [34], (0) no foci, (1) 3 foci, (2) 3–6 foci, (3) 7–12 foci, and (4) > 12 foci/field at low magnification power.

### Assessment of the silver content in the muscle and some vital organs

The amount of silver in muscle and some edible organs like the heart, liver, and spleen tissue was measured using an inductively coupled plasma mass spectrometer (ZEISS, AAS5, and Germany). Briefly, 0.5 g tissue samples were treated with strong nitric acid and 30% H2O2 overnight, then cooked in a microwave digestion system (ETHOS One; Milestone, Sorisole, Italy) until it was totally digested and colorless. The remaining solutions were then diluted with 2% nitric acid after the samples had had time to cool [35].

### Statistical analysis

All data were expressed as mean ± standard error of the mean (SEM) and were analyzed using SPSS version 24.0 software (SPSS Inc., Chicago, IL, USA). One-way analysis of variance (ANOVA) followed by *post-hoc* Duncan’s test was performed to compare the nanoparticle’s treatment groups versus the control group. Whereas the non-parametric values as the histological lesion scoring were expressed as median and were analyzed using the Kruskal Wallis H test followed by the Mann-Whitney *U* test. Values of *P* ≤ 0.05 were considered statistically significant.

## Results

### Characterization of the prepared nanoparticles

The results of the physicochemical characterization of the prepared nanoparticles were illustrated in supplementary materials (Fig. S1, S2). UV-spectroscopy showed the characteristic Surface Plasmon Resonance (SPR) absorption peak at 404 nm with a Gaussian distribution curve for the prepared AgNPs. TEM electrographs showed spherical-shaped particles with a size range of 17 ± 4 nm for both AgNPs and CS/Ag NCs. DLS showed an average particle size of 17 nm with an excellent polydispersity index (PDI) of 0.392 for AgNPs and 20 nm for CS/Ag NCs as well as zeta potential 66 mV for CS/Ag NCs. XRD analysis based on low Bragg’s reflections was used to examine the phase formation of silver nanoparticles. Typical diffraction pattern with distinct peaks at 38.14°, 44.41°, 64.61°, and 77.74° 2 angles, respectively, corresponding to the hill parameters of (111), (200), (220), and (311). The generated diffraction pattern was compared with the installed PDF4 software’s default ICCD library, card number: (04-003-5625). Moreover, chitosan and silver were present in the synthesized CS-Ag NCs, although there were no impurity phases, according to the XRD pattern. Chitosan peak appeared at 2 values of the broad peak between 15 and 35 degrees. The face-centered cubic structure was used to index the silver peak locations, and this structure agrees well with the JCPDS card No.04-004-8730. The three silver peaks that were discovered correspond to the reflections (111), (220), and (311), respectively. The position and relative intensity of all the diffraction peaks of the samples were consistent with the crystalline pattern of silver, proving that the produced nanoparticles were in fact silver nanoparticles.

### The growth performance

A significant increase in body weight gain and a decrease in the food conversion ratio were observed in all nanoparticle-treated birds compared with the control one. Birds given either 0.5- or 5 ppm CS/Ag NCs showed marked improvement in BWG and FCR compared with those receiving AgNPs (Table 1).

**Table 1:** The effect of AgNPs and CS/Ag NCs on chicken body weight and FCR

	Control	0.5 AgNPs	5 AgNPs	0.5 CS/Ag NCs	5 CS/Ag NCs
Week 1					
BWT	147 ± 5.2^a^	125 ± 9.2^b^	120 ± 12.2^b^	134 ± 7.2^ab^	138 ± 6.2^ab^
FCR	1.4 ± 0.2^a^	1.3 ± 0.5^a^	1.5 ± 0.2^a^	1 ± 0.1^b^	0.9 ± 0.5^b^
Week 2					
BWT	406 ± 10.2^a^	382 ± 19.2^a^	372 ± 17.6^a^	385 ± 15.2^a^	370 ± 18.4^a^
FCR	1.7 ± 0.5^a^	1.6 ± 0.4^a^	1.7 ± 0.2^a^	0.9 ± 0.2^b^	0.8 ± 0.5^b^
Week 3					
BWT	719 ± 15.2^a^	683 ± 40.2^a^	638 ± 39.6^a^	722 ± 36.2^a^	819 ± 29.4^c^
FCR	1.9 ± 0.3^a^	1.8 ± 0.1^a^	2 ± 0.5^a^	0.8 ± 0.1^b^	0.9 ± 0.4^b^
Week 4					
BWT	1666 ± 35.3^a^	1200 ± 45.1^b^	1187 ± 50^b^	1777 ± 55.2^a^	1830 ± 53.4^c^
FCR	1.8 ± 0.4^a^	1.8 ± 0.3^a^	1.9 ± 0.5^a^	1.2 ± 0.3^b^	1.3 ± 0.2^b^
Week 5					
BWT	1785 ± 56.1^a^	1550 ± 60.2^b^	1599± 70.6^b^	1944 ± 75.2^c^	1995 ± 77.4^c^
FCR	1.9 ± 0.5^a^	1.8 ± 0.1^a^	2.1 ± 0.5^b^	1.1 ± 0.2^c^	1.3 ± 0.2^c^

Values represented as mean ± SEM (n = 10 birds/group). Different superscript letters (a-c) mean significant difference at *P ≤ 0.05*

### Behavioral assessment

The behavioral results in Table 2 show a significant increase in feeding and drinking behaviors in the group receiving 0.5- or 5 ppm CS/Ag NCs compared with the broiler in other groups. Furthermore, the percentages of leg and wing stretch, as well as preening, were higher in birds fed with CS/Ag NCs either at 0.5- or 5 ppm when compared to those fed with AgNPs and control groups.

**Table 2:** The effect of AgNPs and CS/Ag NCs on some behavioral parameters

	Control	0.5 AgNPs	5 AgNPs	0.5 CS/Ag-NCs	5 CS/Ag-NCs
Feeding %	24.6 ± 1.2 ^a^	24.4 ± 1 ^a^	24.2 ± 0.7 ^a^	33 ± 0.7^b^	35.5 ± 0.6^b^
Drinking %	5.6 ± 1 ^a^	5.4 ± 0.6 ^a^	5.2 ± 0.5 ^a^	9 ± 0.7 ^b^	10.2 ± 1.2 ^b^
Wing stretch %	2.6 ± 0.6 ^a^	2.6 ± 0.4 ^a^	2.4 ± 0.2 ^a^	5.6 ± 0.5 ^b^	6.6 ± 0.4 ^b^
Leg stretch %	1.8 ± 0.2 ^a^	1.6 ± 0.4 ^a^	1.4 ± 0.2 ^a^	3.2 ± 1.3 ^b^	3.4 ± 01.3 ^b^
Preening %	10 ± 0.7^a^	9.8 ± 0.6 ^a^	9.8 ± 0.4 ^a^	13.2 ± 0.6 ^b^	15.2 ± 0.3 ^b^

Values represented as mean ± SEM (n = 10 birds/group). Different superscript letters (a-c) mean significant difference at *P ≤ 0.05*

### The antibody titers against ND and AI virus

**Fig. 1:**
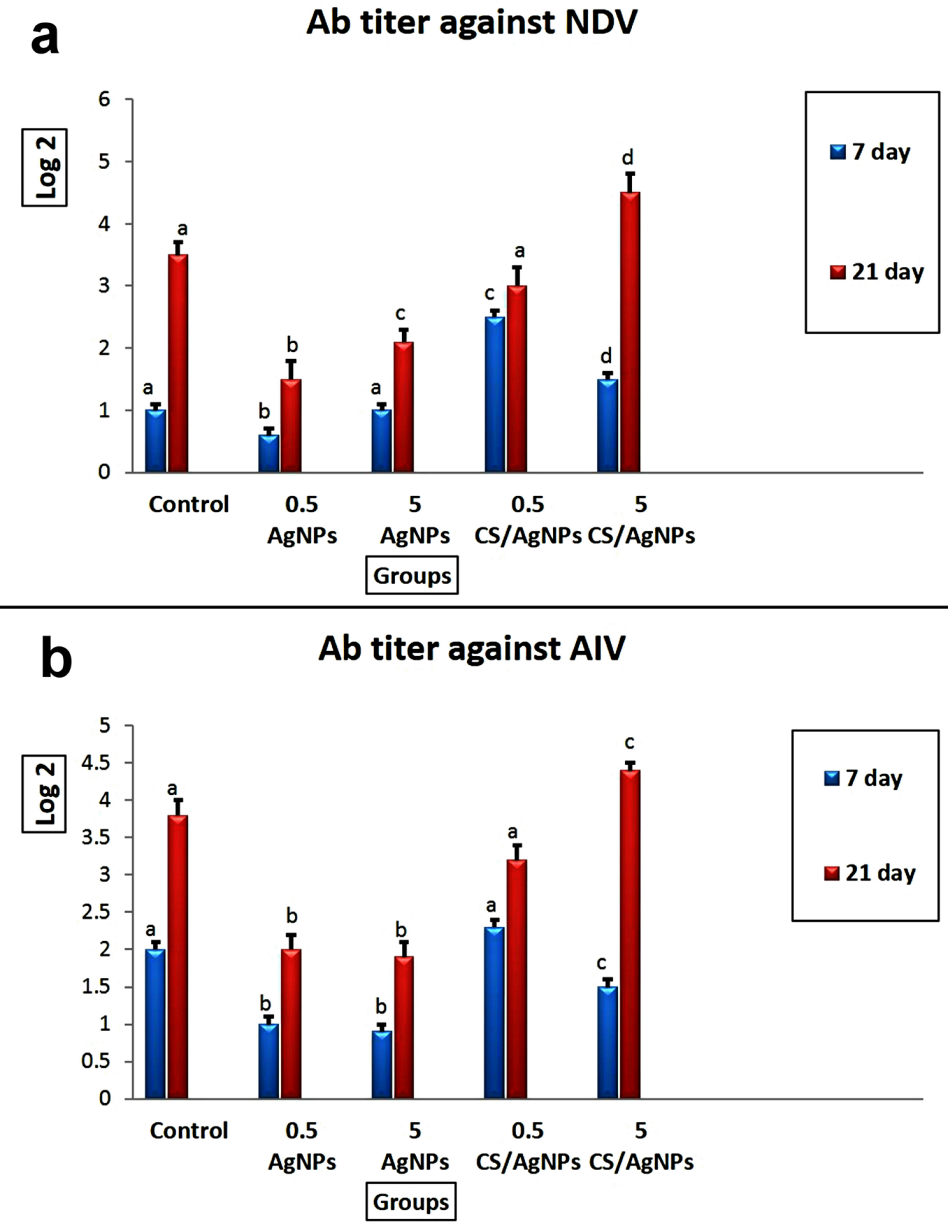
The effect of the nanoparticles on the antibody titers against ND and AI virus in broiler chicken. Values represented as mean ± SEM (n = 10 birds/group). Different superscript letters (**a**-**c**) mean significant difference at *P* ≤ 0.05

Compared with the control group, all AgNPs treated birds didn’t exhibit any significant difference in the antibody titers against ND and AI virus. Otherwise, the group receiving CS/Ag NCs either at 0.5- or 5 ppm showed a significant increase in the antibody titers against ND and AI virus compared with other groups, and the highest level recorded in the group receiving 5 ppm CS/Ag NCs (Fig. 1).

### Oxidative stress evaluations

**Fig. 2:**
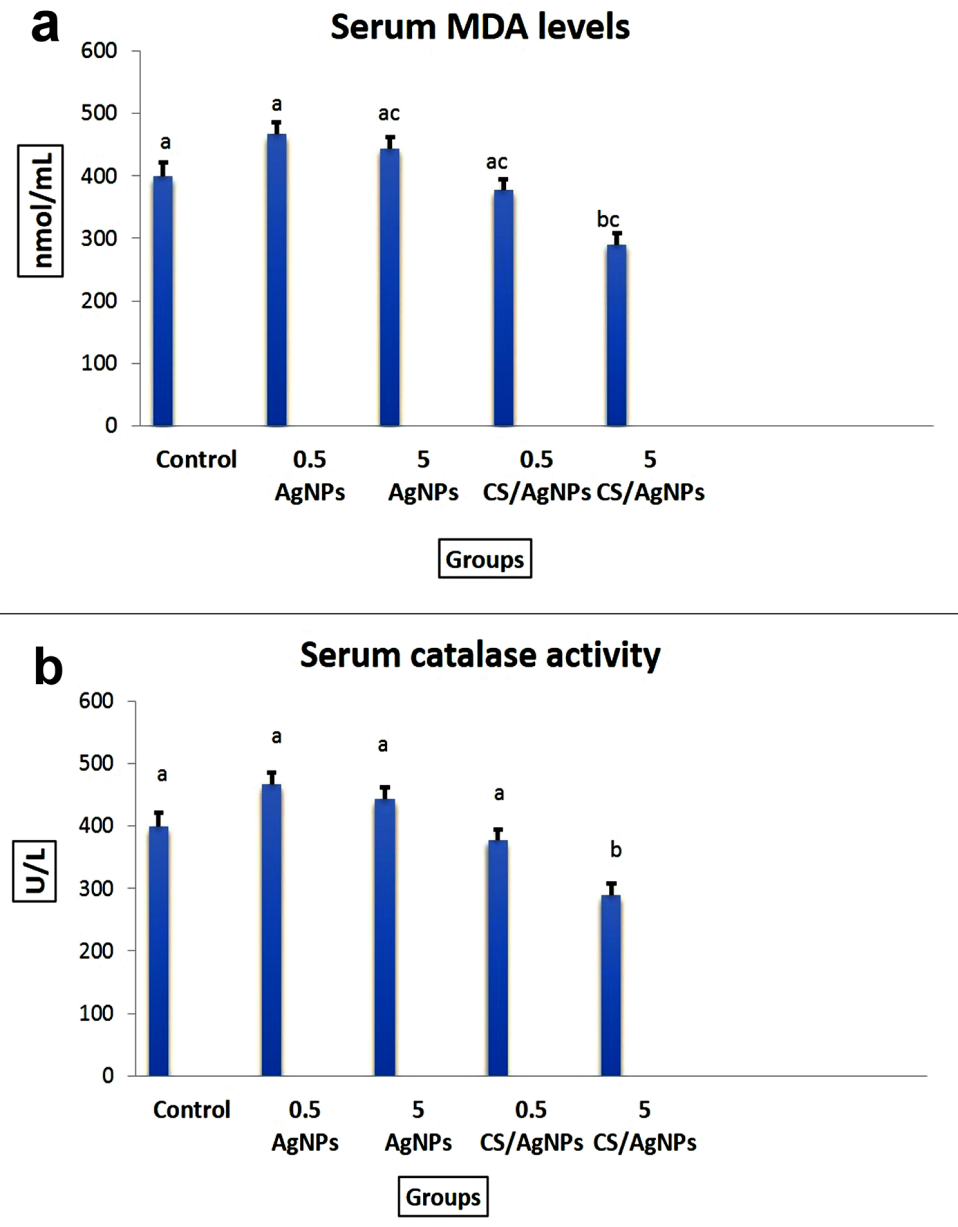
The effect of the nanoparticles on some oxidative stress biomarkers in the serum of broiler chicken. Values represented as mean ± SEM (n = 10 birds/group). Different superscript letters (**a**-**c**) mean significant difference at *P* ≤ 0.05

A significant increase in MDA levels and decrease in TAC and CAT activity were observed in birds that were given 5 ppm AgNPs followed by those receiving 0.5 ppm AgNPs compared with the control group. Otherwise, the group receiving 0.5- or 5 ppm CS/Ag NCs showed marked improvement in the parameters in contrast to other groups (Fig. 2).

### Microscopical findings of different organs

**Fig. 3:**
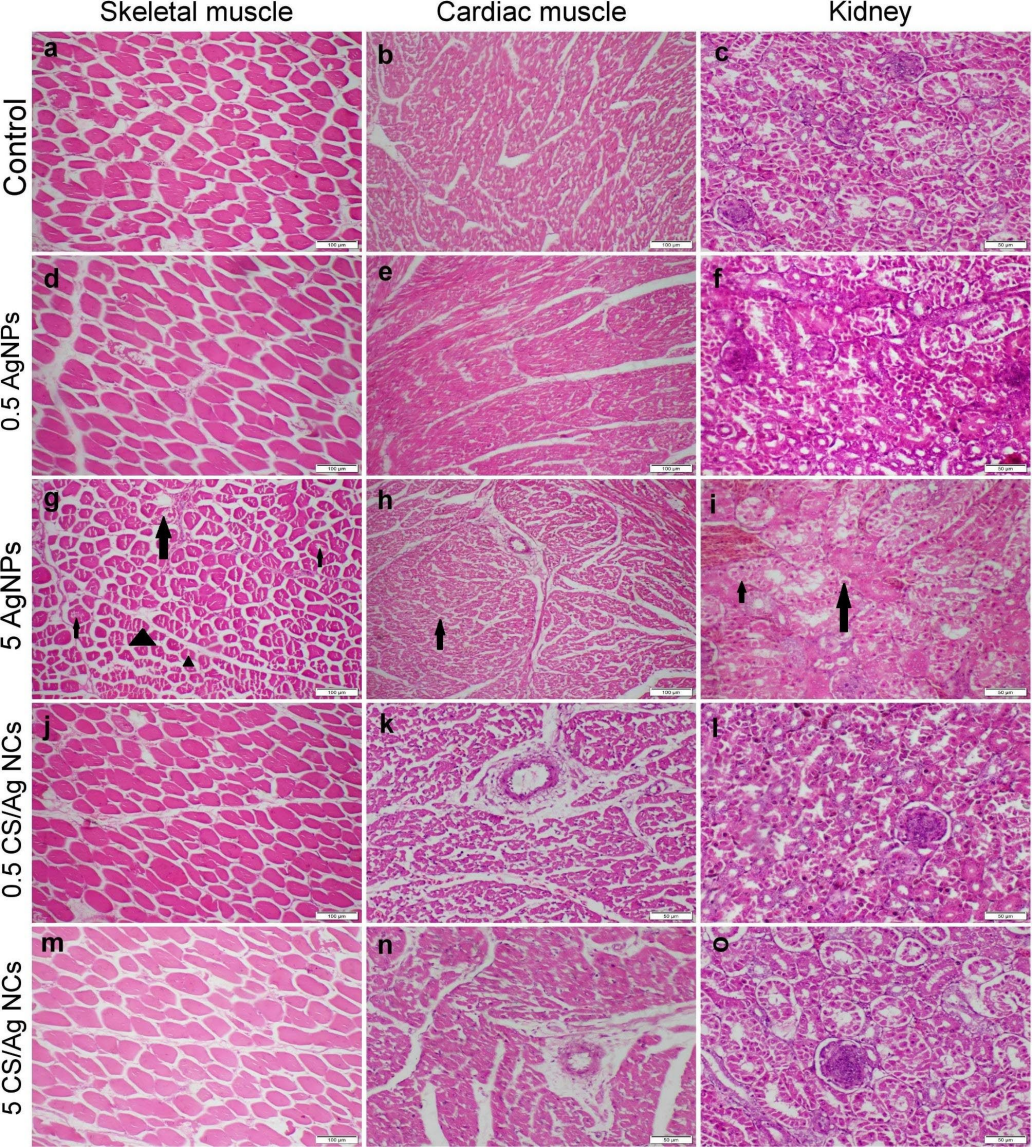
Photomicrograph of skeletal and cardiac muscles and kidney tissue sections stained with H&E representing different treatment groups. (**a**-**c**) Control group showed normal histological structure. (**d**-**f**) Group receiving 0.5 mg AgNPs showed normal histological structure. (**g**-**i**) Group receiving 5 mg AgNPs displayed moderate cellular degeneration and necrosis (arrows) along with splitting of myofibrils (triangles). (**j**-**l**) and (**m**-**o**) Groups receiving 0.5- and 5 mg CS/Ag NCs, respectively, exhibit normal histological organization. Images with scale bar 100 μm were captured at magnification power 10x, whereas images with scale bar 50 μm were captured at magnification power 20x

Skeletal and cardiac muscle sections as well as kidney tissues from the control group displayed typical histological architecture (Fig. 3a-c). Likewise, the group receiving 0.5 ppm AgNPs exhibits normal histological organization (Fig. 3d-f). Otherwise, the group receiving 5 ppm AgNPs demonstrated moderate to severe histological changes. Both skeletal and cardiac muscles showed moderate cytoplasmic vacuolization of some myocytes (Fig. 3g, h). Some sections showed inflammatory cells infiltration between muscle bundles. Kidney sections displayed moderate tubular epithelial degeneration and necrosis along with vascular congestion (Fig. 3i). Furthermore, those obtained from CS/Ag NCs showed normal histological structures (Fig. 3j-o).

**Fig. 4:**
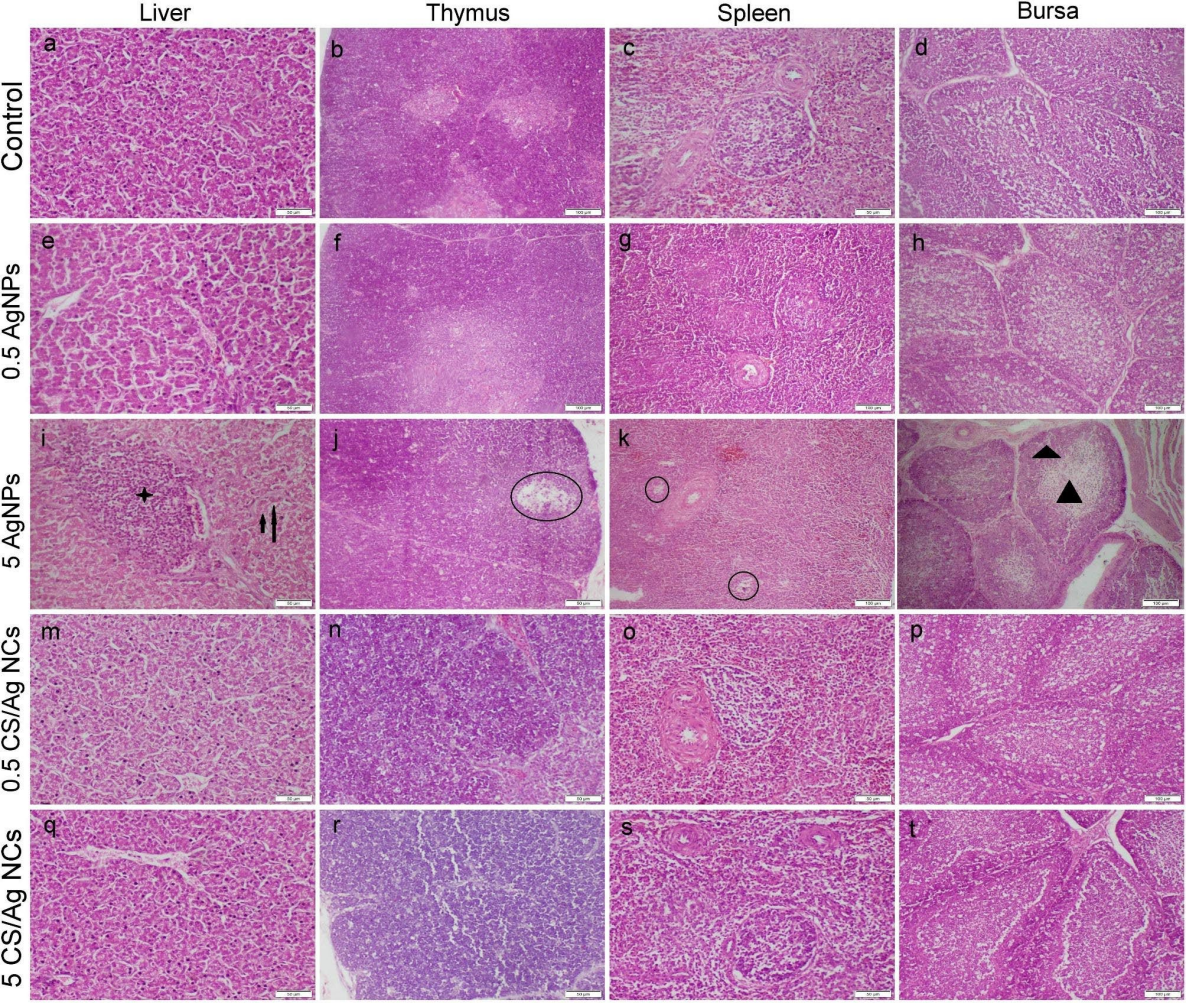
Photomicrograph of tissue sections obtained from different immune organs that stained with H&E representing different treatment groups. (**a**-**c**) Control group showed normal histological structure. (**d**-**f**) Group receiving 0.5 mg AgNPs showed normal histological structure. (**g**-**i**) Group receiving 5 mg AgNPs displayed moderate to severe histological alterations including, cellular vacuolation and necrosis (arrows), inflammatory cells infiltration (star), focal lymphocytolysis (circles), lymphocytic cell depletion (triangles). (**j**-**l**) and (**m**-**o**) Groups receiving 0.5- and 5 mg CS/Ag NCs, respectively, exhibit normal histological organization. Images with scale bar 100 μm were captured at magnification power 10x, whereas images with scale bar 50 μm were captured at magnification power 20x

Regarding immune organs, the control group showed normal histology of the liver, thymus, spleen, and bursa of Fabricius (Fig. 4a-d). Additionally, those obtained from birds receiving 0.5 mg AgNPs displayed a normal microscopic appearance, too (Fig. 4e-h). Otherwise, birds receiving 5 mg AgNPs noticed severe portal and centrilobular infiltration of mononuclear inflammatory cells along with random hepatocellular vacuolation and necrosis in most liver sections (Fig. 4i). Thymus showed a decrease in the cortical layer width with focal necrotic area (Fig. 4j). Lymphoid follicles of both splenic white pulp and bursal tissue exhibit extensive lymphoid depletion and lymphocytolysis associated with enormous number of tangible-like macrophages (Fig. 4k, l). Moreover, administration of CS/Ag NCs at two dosage levels didn’t influence any histological changes in the immune organs (Fig. 4m-t).

Table 3 summarizes the microscopic lesion scoring in all the examined organs. The highest scores of all histopathological parameters were observed in the group given 5 mg AgNPs followed by those receiving 0.5 mg. Meanwhile, the groups’ given CS/Ag NCs either at dosage levels 0.5- or 5 mg didn’t record any significant difference compared with the control group, except for moderate muscular degeneration observed in the 5 mg CS/Ag NCs receiving group.

**Table 3:** Microscopic lesion scoring in different examined organs

	Control	0.5 AgNPs	5 AgNPs	0.5 CS/Ag NCs	5 CS/Ag NCs
**Skeletal and cardiac muscles scoring**					
**Vacuolation**	0^a^	2^b^	2^b^	0^a^	2^b^
**Necrosis**	0^a^	2^b^	2^b^	0^a^	0^a^
**Liver and kidney tissue scoring**					
**Degeneration**	0^a^	3^b^	4^c^	0^a^	0^a^
**Necrosis**	0^a^	2^b^	2^b^	0^a^	0^a^
**Hemorrhage**	0^a^	0^a^	3^b^	0^a^	0^a^
**Inflammation**	0^a^	2^b^	3^c^	0^a^	0^a^
**Thymus, spleen, and bursal tissue scoring**					
**Lymphocytosis**	0^a^	2^b^	4^c^	0^a^	0^a^

Values represented as mean ± SEM (n = 10 birds/group). Different superscript letters (a-c) mean significant difference at *P* ≤ 0.05

### Silver content in the muscle and some edible organs

A significant elevation in silver concentration of the liver was recorded in groups receiving AgNPs at both doses, whereas the group receiving 5 mg AgNPs only displayed a significant elevation of silver contents in broilers’ musculatures compared with those receiving 0.5 mg. However, the silver concentrations of the muscle, liver, spleen, and heart did not significantly differ between the groups receiving CS/Ag NCs and the control group (Table 4).

**Table 4:** Silver contents (ppp) in muscle and edible organs

	Control	0.5 AgNPs	5 AgNPs	0.5 CS/Ag NCs	5 CS/Ag NCs
**Muscle**	0.00 ± 0.0^a^	0.018 ± 0.0^a^	0.02 ± 0.0^a^	0.002± 0.0^a^	0.00 ± 0.0^a^
**Liver**	0.00 ± 0.0^a^	25.00 ± 2.8 ^b^	32.3 ± 1.4^c^	0.08 ± 0.1^a^	0.04± 0.0^a^
**Heart**	0.00 ± 0.0^a^	0.002 ± 0.0^a^	0.001± 0.0 ^a^	0.002± 0.0^a^	0.00 ± 0.0^a^
**Spleen**	0.00 ± 0.0^a^	2.6 ± 0.3^b^	4.1 ± 0.5^c^	0.002 ± 0.0^a^	0.00 ± 0.0^a^

Values represented as mean ± SEM (n = 10 birds/group). Different superscript letters (a-c) mean significant difference at *P* ≤ 0.05

## Discussion

Nanotechnology is a promising as well as emerging technology that will have an essential role in poultry and animal nutrition in the future [36]. Nanoparticles have a variety of benefits, including the ability to enter the cells faster through the stomach’s wall than bigger particle sizes. In addition, nano-additives may be added to natural feed ingredients like protein capsules, micelles, and other components [9]. There are various kinds of nanoparticles particularly AgNPs usually viewed as antimicrobial agents [37]. However, the use of chitosan coating silver nanoparticles as growth promoter potential is not well studied in the broilers, hence our research was intended to evaluate and compare the potential impacts of two different doses of AgNPs and CS/Ag-NCs on the growth performance, oxidative stress damage, immunological indices, and histopathological organizations in different organs of the broilers.

The results of the current research showed that the oral supplementation of chicken with 0.5 and 5 ppm CS/Ag-NCs once a week for 36 days significantly increased the body weight gain (BWG) and decreased the food conversion ratio (FCR) throughout the entire experiment, whereas a group of birds that were treated with 0.5 and 5 ppm AgNPs showed slight improvements in body weight gain and food conversion ratio compared to the control group. The findings of our study could be attributed to the biological actions of chitosan and silver on intestinal pathogenic bacteria, which also caused enhanced development and growth as nutrient absorption was elevated [38]. Moreover, nanoparticles could improve intestinal mineral uptake and consumption, by increasing their surface area, which would enhance growth performance [39].

Our findings also revealed that CS/Ag NCs markedly improved both physical and psychological broilers welfare which are consistent with previously mentioned findings, CS/Ag-NCs improved body weight gain and food conversion ratio. Feeding, drinking, scratching, foraging, perching, resting, and comforting behavior patterns are among the crucial behavioral parameters that determine broiler welfare [40]. Interestingly, the current findings revealed that the CS/Ag-NCs significantly enhanced feeding activity more than those of the control group, whereas AgNPs supplementation slightly enhanced broiler feeding and drinking behavior. Our findings were in context to Ayman et al., [40] who reported that the supplementation of chitosan to broiler food enhanced feeding activity and body weight gain which was attributed to its bactericidal and immunostimulant activities. Regarding AgNPs, some studies revealed the potential of AgNPs to improve both the feeding and drinking behavior of broilers [41, 42], while others showed that AgNPs didn’t affect feeding and drinking [43]. These variations may be related to many factors, including NPs size and concentration, routes of administration, and duration of exposure. In terms of broiler comfort and body care behavior, several studies have demonstrated that comfort activity, including preening, wing, and leg stretching, wing flapping, and body shaking, is crucial for feather care and body maintenance [44]. Preening regulates lipid levels in the feathers and maintains the viability of the chicken’s skin and plumage [40]. Broilers fed with CS/Ag-NCs displayed a significantly increased activity in preening and stretching of the wings and legs compared to the AgNPs and control groups.

A recent study indicated that CS/Ag-NCs can improve the intestinal absorption of minerals and nutrients and animal health [45]. In our study, the histopathological analysis of the examined organs revealed normal histology in the 0.5- and 5 ppm CS/Ag-NCs received groups, confirming that the broiler was in good health. On the other hand, all the examined organs displayed severe histopathological alterations in the 5 ppm AgNPs group. In this group, the lymphoid follicles of the spleen, thymus, and bursa of Fabricius revealed significant lymphocytolysis and lymphoid cell deficiency. Our results suggest that 5 ppm AgNPs may have an immunosuppressive effect on broiler chickens related to T- and B-lymphocyte depletion. In addition, our findings revealed a significant drop in antibody levels against Newcastle disease (ND) and avian influenza (AI) in the 5 ppm AgNPs group. The phagocyte and lymphocyte are well known to be the principal barriers to nanoparticle invasion of animal cells and tissues. Consequently, it is undoubtedly of great interest to study how AgNPs interact with phagocytic cells, their intracellular absorption, as well as immune cell responses to them. Ognik et al. found that orally administered AgNPs with varying sizes enhanced phagocytosis and leukocyte metabolic activity which may be signs of an inflammatory condition evolving in the organism [46].

Reactive oxygen species (ROS) overproduction induced oxidative stress, rendering cells incapable of performing normal physiological activities [47]. Cellular activity disorders entail lipid peroxidation, modulation of inflammatory processes via signal transduction, as well as modulation of gene expression via transcriptional regulator activation, all of which can have cell death and immunotoxic effects [48–50]. There are numerous oxidative stress indicators, including ROS themselves, which reflect the level of ROS overproduction. However, ROS has a short half-life but is highly reactive. Due to this, evaluating the oxidation target products of oxidative stress, such as lipid peroxidation, oxidative nucleic acid damage, and oxidized proteins is more applicable [51]. In the present research, the group that administered 5 ppm AgNPs had significantly higher blood levels of MDA, a lipid peroxidation byproduct, and significantly reduced CAT and TAC activity, which are antioxidants, those that indicated oxidative stress damage. Since membrane phospholipids are important targets of oxidative stress, lipid peroxidation is the primary parameter evaluated to indicate the presence of free radical damage. Peroxidation of lipids results in a progressive deterioration of cell membrane integrity, disturbance of cellular ion homeostasis, as well as dysfunctional membrane transport [52]. The elevated MDA levels and lowered CAT activities after AgNPs exposure in our work were consistent with Sreelekha et al., who found a marked rise in MDA levels and significantly decreased CAT levels in broiler chicken intestinal mucosa after 42 d of AgNPs exposure [53].

Comparing the group of birds treated with AgNPs to those treated with CS/Ag-NCs, the current study found a significantly higher level of silver in both the liver and spleen of the treated group of birds. Our research suggests that the AgNPs accumulation in broiler chicken livers may be transferred to consumers, causing a range of undesirable adverse effects. These findings are consistent with much previous research that found that broiler chickens’ livers retained Ag significantly more than other organs [54, 55]. Chitosan nanoparticles also serve as a capped site for nanoparticle stability because they have a certain functional group (amino compounds) that binds with Ag ions [56]. Additionally, CS/NCs function as a binding agent for AgNPs by creating a network on the surface of NPs, which enables uniform AgNPs distribution upon the surfaces without any discernible aggregation consequences [57]. Our study provides insights into the ability of CS/Ag NCs to enhance the growth performance and immune status of broiler chickens, however, further studies are required to confirm these insights on other species including layer chickens, pigeons, rabbits, and others.

## Conclusion

Based on our data, we conclude that the oral intake of either 0.5- or 5 ppm CS/Ag-NCs weekly to broiler chickens was beneficial in improving their growth performance, welfare index, and immunological status, which neither left toxic residues in muscles and edible organs nor damaging the histological organizations of their internal organs. However, broiler chickens exposed to 5 ppm AgNPs exhibited severe cytotoxicity as evidenced by changes in oxidant/antioxidant parameters, and histological structures. Consequently, we recommend utilizing 0.5 ppm CS/Ag NCs as feed additives in broiler farms to promote their growth performance and strengthen their immune defense without leaving any residues in the muscle and edible parts of broiler chicken.

### Electronic supplementary material

Below is the link to the electronic supplementary material.

Supplementary Material 1

Supplementary Material 2

## Data Availability

All data generated or analyzed during this study are included in this published article [and its supplementary information files].
