# Supplementary material for: Chitosan coating silver nanoparticles as a promising feed additive in broilers chicken
Source: BMC Vet Res. 2023 Dec 9;19:265. doi: 10.1186/s12917-023-03826-7 (PMC10709949; doi:10.1186/s12917-023-03826-7)
Supplement: Supplementary file 1 — Supplementary Material 1 [file 12917_2023_3826_MOESM1_ESM.docx]

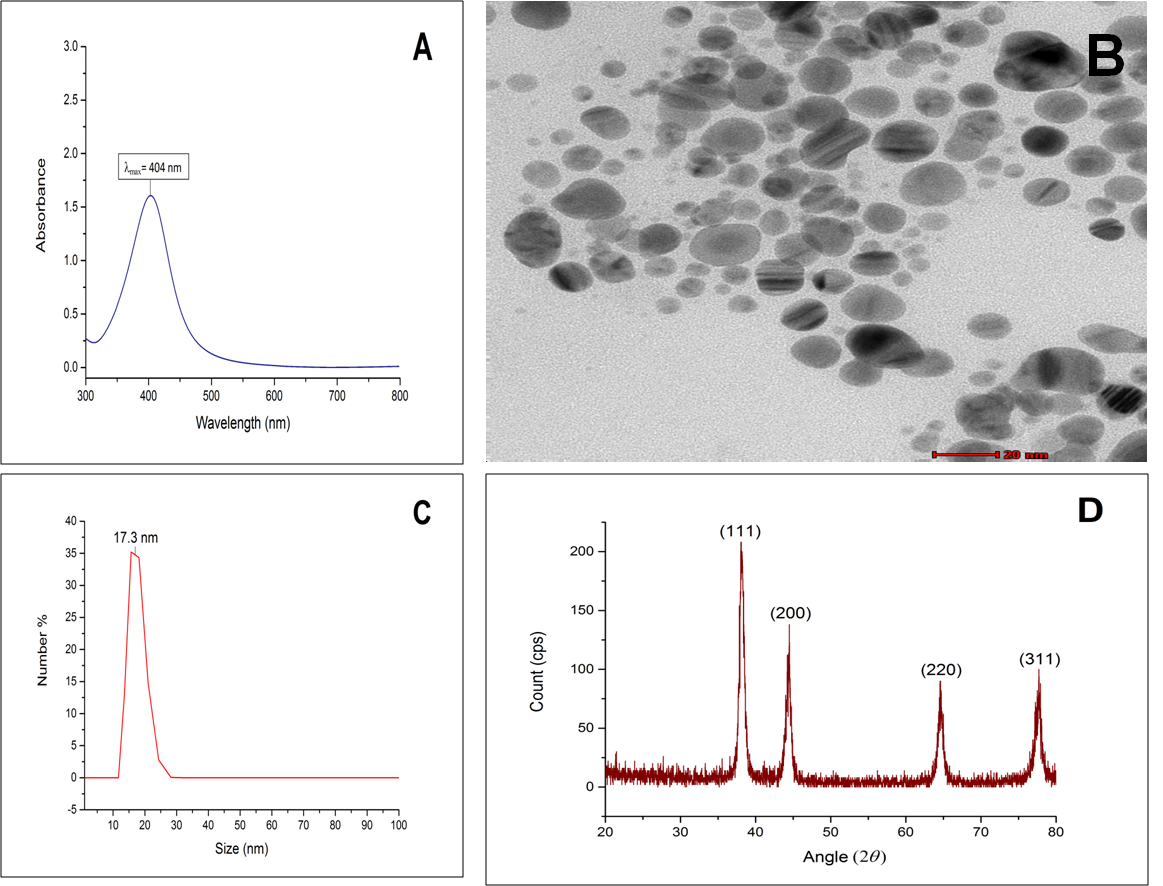


**Fig 1S.** Characterization of silver nanoparticles (AgNPs). **(A):** Absorption spectrum of silver nanoparticles. **(B):** HRTEM image showing spherical shape of prepared silver nanoparticles with average size 17 nm. **(C):** Particle size distribution of prepared silver nanoparticles showing the average size of 17 nm. **(D):** XRD pattern analysis indicating the formation of silver nanoparticles with cubic unit crystal.

| 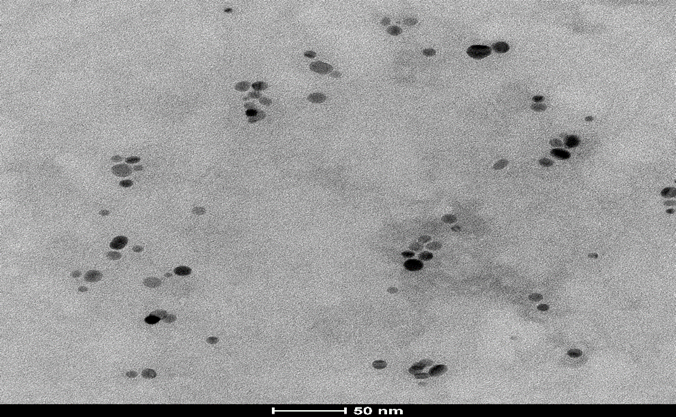  A | 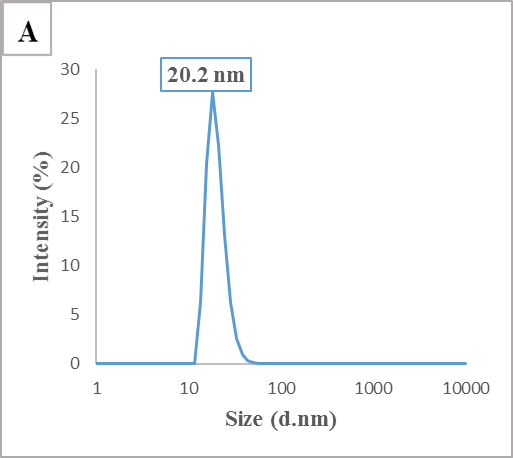  B |
| --- | --- |
| 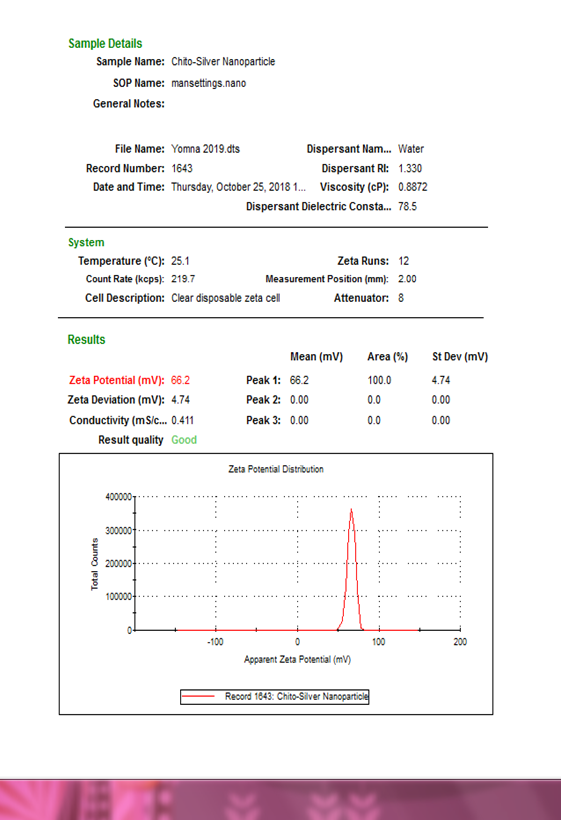 **c**  66.2 nm | 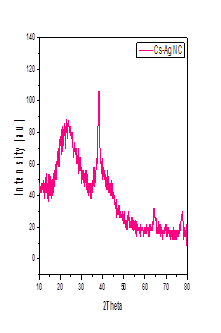  D |

**Fig. 2S.** Characterization of chitosan coating silver nanoparticles (CS/Ag NCs). (A) HR-TEM image showing spherical shaped particles, (B) Particle size distribution curve, (C) Zeta potential and (D) X-ray powder diffraction patterns of CS/Ag NCs.
